# Food Security, Nutritional Supply, and Nutrient Sources in Rural Burkina Faso

**DOI:** 10.3390/nu15102285

**Published:** 2023-05-12

**Authors:** Sakiko Shiratori, Yachiyo Tobita, Eveline M. F. W. Sawadogo-Compaoré

**Affiliations:** 1 Japan International Research Center for Agricultural Sciences (JIRCAS), Ibaraki 305-8686, Japan; 2 Faculty of Life and Earth Sciences, Graduate School of Science and Technology, University of Tsukuba, Ibaraki 305-8573, Japan; s2030239@u.tsukuba.ac.jp; 3 Institute for Environment and Agricultural Research (INERA), Ouagadougou P.O. 01 BP476, Burkina Faso; compeve@yahoo.fr

**Keywords:** Africa, Burkina Faso, food consumption score (FCS), food security, macronutrient balance, micronutrient supply, nutrition, ordered logit, regional difference, seasonality

## Abstract

Rural communities are more likely to encounter constraints in achieving food security and adequate nutritional supply. This study explores food security, nutritional supply, nutrient adequacy, macronutrient balance, recipes, and nutrient sources based on bi-monthly household surveys in rural villages in Northern and Southern Burkina Faso from 2019 to 2020. Food security across time and its quantity dimensions were measured using the food consumption score (FCS). Ordered logit regression showed that FCS was significantly influenced by season, region, and household characteristics such as the head’s education and women’s possession of personal plots. The regional differences were large: Households categorized as having “poor” diets were approximately 1% and 38% in the south and north, respectively. Nutrient adequacy was assessed by converting 24 h dietary recall into nutrient supply and comparing the results to the requirement. While macronutrient balance appeared adequate in the pooled sample, it became unacceptable when the two regions were considered separately. Most micronutrient supplies were insufficient. Cereals were the main nutrient sources, and leaves of crops and potash (additive containing potassium) were also non-negligible for micronutrient supplies. Overall, we found large regional differences in nutrition and food security, indicating that various local contexts must be considered for effective nutritional improvement.

## 1. Introduction

Ensuring food and nutrition security is one of the key global agendas for sustainable development. According to [1], globally in 2021, 702–828 million and 2.3 billion people faced hunger and moderate or severe food insecurity, respectively. Hunger prevalence in Africa is the highest; both the percentage and number of hungry people on the African continent have been rising. Additionally, Africa has the highest prevalence of moderate or severe food insecurity, reaching 57.9% in 2021; this is approximately twice the world average of 29.3%, and is on an upward trend. Furthermore, nutrition inequalities exist across and within countries, primarily affecting the most vulnerable groups [2].

In ensuring food security and adequate nutritional supplies, people in rural areas may encounter several constraints such as income, market access, climate conditions, or seasonal availability. Stability—meaning people having access to adequate food at any time—is an important aspect of food security; it comprises both the availability and access dimensions of food security. When considering nutritional supply, it is necessary to include both energy and micronutrient supplies. Macronutrients are sources of energy; an imbalance of carbohydrates, proteins, and fat can increase the risk of several chronic diseases [3]. Micronutrients are also important for living a healthy life. Micronutrient deficiencies prevent the normal functioning of the immune system, growth, and reproduction (for instance, iron deficiency leads to anemia, whereas vitamin A deficiency leads to blindness). Many African countries face specific nutrient deficiencies, despite normal or less-than-normal calorie intake [4].

Among the African countries burdened heavily with malnutrition, this study focuses on rural households in Burkina Faso. Burkina Faso is a landlocked country located in West Africa. In Burkina Faso, 30.5% of the population lived below the global poverty line at $2.15/day in 2018, and 68.8% lived in rural areas in 2021 [5]. Burkina Faso is afflicted with several food security and nutritional challenges. In 2020, more than half the population (52.6%) was moderately or severely food insecure, of which 18.5% were severely food insecure. The prevalence rate of anemia among women of reproductive age, which is related to micronutrient deficiencies such as iron, was also more than half (52.5%) in 2019 [5]. The Direction of Nutrition was created in the Ministry of Health in 2002 and it has taken leadership on the nutritional agenda since then. It advocated and prioritized the nutritional agenda and coordinated intersectoral collaboration, which contributed to nutritional improvement in Burkina Faso; however, there has been difficulty in ensuring funding and human resources at the decentralized level [6]. In addition, political instability, emerging conflicts and displacement, climate change, and rapid population growth have been challenges for food security and nutrition in Burkina Faso.

For agriculture in Sub-Saharan Africa, which heavily depends on being rainfed and is dominated by a smallholder farming system with limited investment, climate factors such as precipitation and temperature are critical to ensure regional food security [7]. The climate in Burkina Faso varies vastly according to longitude. From north to south, three climatic zones exist: Sahelian, Sudan–Sahelian, and Sudanian. In addition to climate, there are regional differences in culture and customs among the various ethnic and language groups, religions, and geographic locations [8]. As the situation of food security differs by region [9] and geographical disaggregation is necessary to assess regional food and diet diversity [10], we compared two climatically and culturally different regions from the north and south of the country.

Seasonal fluctuations due to harvesting times or price shifts have also been recognized as a critical factor influencing stability, particularly in sub-Saharan Africa. Burkina Faso’s major food crops are maize, millet, and sorghum, for which harvest seasons are from August to December. Accordingly, June–August is considered the lean period [11]. Studies show that due to market availability issues, poor urban households in Burkina Faso change their food sources according to the season, which lowers nutrient intake in the lean season compared with the post-harvest season [12]. In rural areas, women shift their food consumption in the post-harvest season, resulting in a higher intake of most micronutrients, except for vitamin A. Vitamin A intake is lower in the post-harvest season, due to the consumption and availability of vitamin A-rich dark green leafy vegetables during the lean season [13].

The objective of this study is to explore food security, nutritional supply, and nutrient sources in rural households in Burkina Faso. Food security across the time and quantity dimensions of nutrient supply is measured using the food consumption score (FCS). We also investigate the factors that influence FCS by applying ordered logistic regressions. Then, we explore the quality dimensions of nutrition by converting 24-h recall data into nutrient supply. To determine food groups that contribute to specific nutrients, we also examine nutrient sources. We use bi-monthly data that can capture slight seasonal changes in detail, compared to previous studies that merely compare the situation of pre- and post-harvest. By understanding rural farmers’ food consumption and nutritional supply through our original field interview surveys in different regions in Burkina Faso, we aim to contribute to ensuring food security—including appropriate nutrient supply—in a realistic way.

## 2. Materials and Methods

We conducted a series of interview surveys with randomly selected households in rural Burkina Faso from April 2019 to February 2020. To account for regional differences, we selected two communes from the north and south of the country. The north commune, Yako, is approximately 109 km northwest of Ouagadougou, the capital of Burkina Faso. The south commune, Po, is located 148 km south of Ouagadougou. For this study, we selected three rural villages in Yako (Gobila, Gollo, and Taonsgho) and three rural villages in Po (Pinyiri, Torem, and Adongo) as their populations, distances from the main road, and distances from the city center were comparable.

To establish a complete list of households, we conducted a census survey of all households located in these six villages in January 2018, before the interview survey started. The total number of households in these six villages was 625. From the 625 households on our list, we randomly selected 230 target households without changing the proportion of households selected from each village. For these target households, we conducted an interview survey at six different times during the study period, in two-month intervals. Interviewers repeatedly visited the households to conduct face-to-face interviews, carrying a tablet terminal on which the pre-tested structured questionnaire was implemented. There were occasions when the interviewers found the residents unavailable, or when they were unable to obtain valid answers after several attempts, due to seasonal relocation, road conditions, health reasons, or refusal. The total number of interviews analyzed in this study was 1176.

We collected information on the households’ demographic backgrounds, dietary habits, agricultural production, agricultural field size, and food consumption, among other parameters. For diet- and food-related questions, we attempted to speak to the person primarily responsible for food preparation in the household. When they were unavailable, we questioned the secondary person responsible for food preparation. Based on the data from these six survey rounds, including seven-day food consumption and 24-h recall, we explored food consumption patterns and nutritional supplies such as FCS, nutrient supply, and food group contributions to nutrient supply. For seven-day food consumption, we collected information on how many days each food group was consumed in the past seven days. In contrast, in 24-h recall, we obtained more detail about their food habits such as the occasion, timing, recipes, ingredients and their proportions, members who eat the meals, and leftovers.

FCS is a composite score in terms of dietary diversity, food frequency, and the relative nutritional importance of different food groups [14]. To calculate FCS based on the household’s consumption of eight different food groups over the previous seven days, the consumption frequencies were aggregated and multiplied by their weight (Table 1). The FCS ranges from 0 to 112. Applying the World Food Program’s recommended cut-offs, the FCS can classify households into three categories of food consumption status based on the following thresholds: “poor” (0–21), “borderline” (21.5–35), or “acceptable” (>35). To test whether the observed distribution of FCS was independent of seasonal changes, we performed a Pearson’s chi-squared test for independence. Moreover, as these three FCS categories are ordinal, we further performed ordered logit regression to explore the factors that influence the FCS category to which a household’s diet belongs, thus allowing intragroup correlations among households.

The FCS is useful for categorizing and tracking households’ food security across time, especially as a proxy for the caloric sufficiency of food security [15]. Hence, we applied this index to analyze food security across time, and the quantity dimension of food security. The FCS can also be used to assess nutrient quantity; however, there is an ongoing debate on whether it can be used to evaluate nutrient quality. The FCS does not provide accurate information on nutritional intake, implying that two populations with the same FCS could have different nutritional intake levels. It can capture nutritional quality approximately [10] but it has not been validated for this purpose [16].

Because the one-week food consumption information used to construct FCS just contained the categories without considering the amount, we further calculated the nutrient intake per capita based on 24 h recall data. We asked interviewees to recall all food and drink consumed in the previous 24 h at the household, including the quantity of all ingredients used in the recipes, how many household members ate the food, and the ratio of leftovers. We measured the weight in grams of the usual units (such as one plate) and used the data to convert quantity units to grams. We could not obtain detailed information on the food consumed away from home; therefore, we calculated per capita food consumption by dividing household food consumption at home by the number of household members who shared the food. We also adjusted the quantity according to edible portions and leftovers.

To calculate per capita nutrient supply, food consumption was converted to nutrient supply using the West African Food Composition Table [17]. The West African Food Composition table presents the average values of the compositional food data collected from nine West African countries (Benin, Burkina Faso, Gambia, Ghana, Guinea, Mali, Niger, Nigeria, and Senegal). The National Nutrient Database for Standard Reference Legacy Release [18] was used for conversion to fill in some blanks. After calculating the nutrient supply per person per day, we explored the regional differences and excess/deficiency of nutrient supply. To view the regional differences, we employed the Mann-Whitney U test after a skewness test confirmed that nutrient supply was not considered to be normally distributed.

Nutritional requirement information is necessary to assess nutrient supply adequacy. Nutritional requirements depend on individual characteristics, such as age, sex, physical activity level, and body weight. For simplicity, we used the requirements of the representatives as a reference. The reference was defined as follows. Participants’ average age was approximately 24 years, and the gender balance was almost equal. To calculate protein requirement, which is based on body weight, we referred to a national survey that assigns 65.2 kg for males and 59.0 kg for females as national averages [19]. Hence, we set a 24-year-old 65 kg male and a 24-year-old 59 kg female as representatives and took the average of the two.

To establish a benchmark for nutritional requirements, we applied the estimated average requirement (EAR), which is the nutrient quantity estimated to meet the requirement for half the healthy individuals in the life stage and gender groups. However, the EAR is not appropriate for use as an energy requirement. Instead, to assess the average individual energy requirement, we used the Average Dietary Energy Requirement (ADER), as it is an appropriate normative reference for adequate nutrition in the population [20]. We used the ADER of Burkina Faso for 2017–2019 [21], which considers country differences due to differences in the population’s sex-age composition and attained height.

The acceptable macronutrient distribution range (AMDR) is expressed as a percentage of total energy intake. If all main energy sources (carbohydrates, proteins, and fat) contribute to energy intake within the AMDR, it is associated with a reduced risk of chronic disease. The AMDR is 45–65% for carbohydrates, 20–35% for fat, and 10–35% for protein, of the total energy intake for adults [5]. We calculated the percentage of energy produced by the macronutrients by multiplying the protein supply (in grams) by 4 and the fat supply (in grams) by 9; the rest was considered as the energy generated from carbohydrates.

We also investigated the contribution of each food group to nutrition, based on nutrient supply converted from the 24-h recall. We illustrated the nutrient sources for each nutrient by classifying seven food groups as cereals, roots/tubers, legumes, fruits/vegetables, meat/fish/eggs, fat/oil, and others. Although the FCS used eight food categories by definition, we combined fruits and vegetables as one group, as fruit consumption was very small in this sample. Among many nutrients, we selected to examine the intake of energy, macronutrients (protein, carbohydrate, and fat), and several important essential micronutrients (calcium, iron, zinc, vitamin A, thiamin, riboflavin, niacin, vitamin B6, folate, and vitamin B12).

This study involved human participants, but it was not an intervention study. No medical devices were used, and no invasive procedures were conducted. All studies were based on face-to-face interviews that gathered data related to household demographics, agricultural production, food consumption, and dietary recall. Informed consent was obtained verbally from all participants at the beginning of each survey. Participants were made aware that they were not obliged to complete the survey and could withdraw at any time. This study was authorized by the Centre National de la Recherche Scientifique et Technologique, Burkina Faso (N° 2020-MESRSI/SG/CNRST/DG/DGA-RC), which stated obtaining ethical approval for this study was not considered necessary. All data were anonymized with no personal identification information retained and stored securely.

## 3. Results

### 3.1. Descriptive Statistics of the Households

Table 2 presents the descriptive statistics of the households by commune (Yako and Po) and overall. Many indicators in Po and Yako were dissimilar, which could be associated with the geographical positions of these two communes. Gobila, Gollo, and Taongsho belong to Yako (north), and Pinyiri, Torem, and Adongo belong to Po (south). The household size presents the average number of household members across the rounds. The under-five child ratios are calculated as the number of children under five years in the household, divided by household size.

The statistics of the household heads are presented to show the households’ characteristics. Most household heads were men. The ethnicity of the head was quite different between the two regions, such that all the household heads in Yako were Mossi and more than three-quarters of the household heads in Po were Gouroussi. The religion of the household heads also showed regional differences: For instance, the ratio of Christians was higher in Yako than in Po, and that of Muslims was higher in Po than in Yako. A majority of household heads had never attended school. Various languages are spoken in Burkina Faso, and many people understand multiple languages. In our sample, all household heads in Yako understood Mossi, with Gouroussi/Kassena being the language most understood among household heads in Po.

Women’s plot indicates whether households had a women’s plot in addition to the family plot. Women’s plots are relatively small plots where women take control and make decisions on what to grow. Field size indicates the total size of cropping fields possessed by each household, including the size of women’s plots. Households in Po tended to have larger fields. From the number of crops grown in 2019, we can see that the households in Po tended to grow a greater variety of crops.

### 3.2. Food Consumption Scores (FCS)

**Figure 1:**
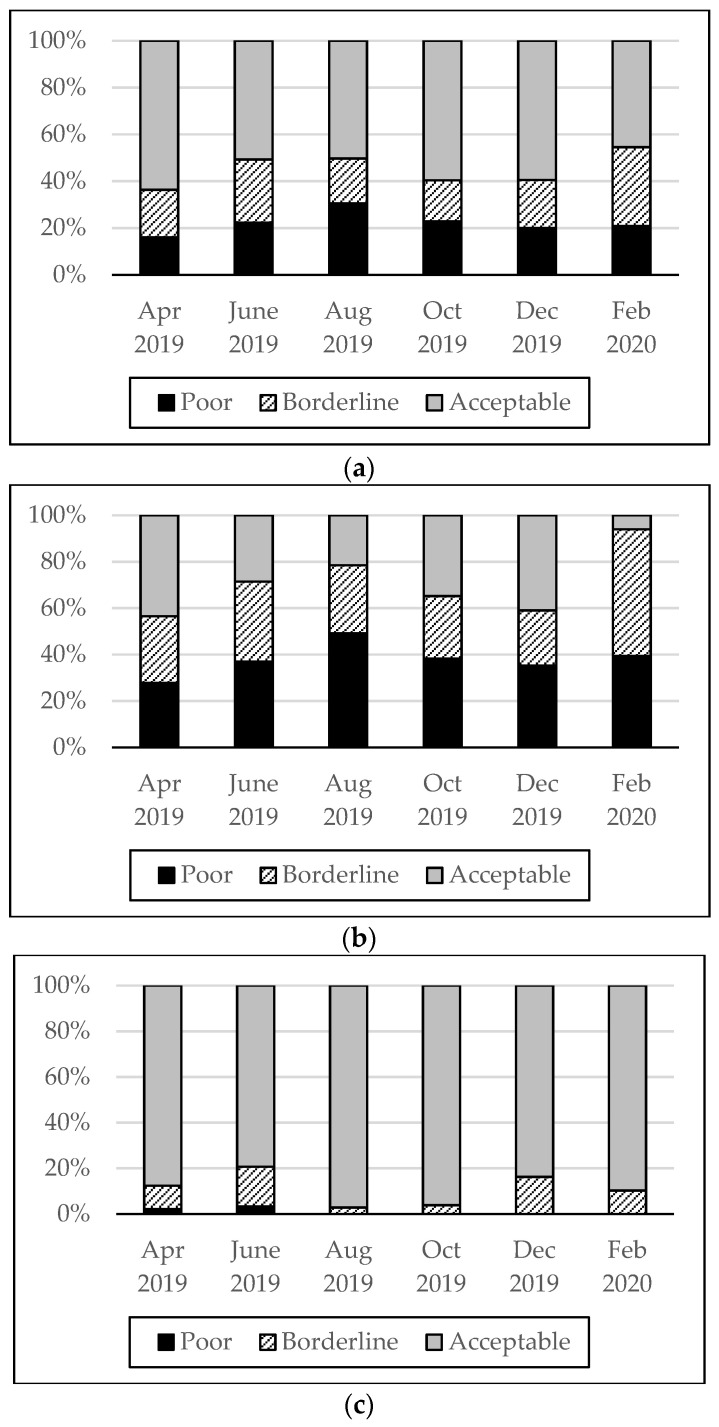
Percentage of households categorized by FCS for (**a**) pooled sample, (**b**) households in Yako, and (**c**) households in Po.

Figure 1 illustrates the ratio of households categorized by the FCS cut-off point in each survey round from April 2019 to February 2020, classified for each commune and pooled sample. From the FCS, the households’ diets were categorized as poor (20%), borderline (20%), and acceptable (60%) in the pooled sample. However, one striking finding is the regional gap. Approximately 38% of the households in Yako were categorized as having a “poor” diet. In contrast, more than 90% of Po’s households’ diets were categorized as acceptable, with only 1% falling into the “poor” category at the annual average.

Figure 1 also shows seasonal fluctuations of the FCS index by survey rounds. Pearson’s chi-squared test’s *p*-value was 0.000 in the pooled samples (0.000 for Yako and 0.005 for Po), which suggests that FCS and seasonal change are not independent of each other. In the pooled sample, the ratio of households placed in the poor dietary group was highest in August, reaching 30%. As mentioned, this is the lean season before the main harvest starts in autumn. This suggests that food security status across time could be affected by seasons, such as lean (pre-harvest) and post-harvest seasons.

Furthermore, we conducted ordered logit regressions to see the factors influencing FCS. The results are presented in Table 3. The dependent variable is the FCS level with three possible ordered values of 1 (poor), 2 (borderline), and 3 (acceptable). To see the seasonal effect clearly, we grouped six rounds into three season dummies as lean (June and August), middle (February and April), and post-harvest (October and December). Season significantly affects FCS, showing a decreasing score with time after the harvest. The commune, whether Yako or Po, has the largest absolute value of log-odds estimate in this model and is the most influential factor to determine FCS categories. The ordered log-odds estimate for households in the higher FCS category in Yako is 3.11 less than those in Po, when the other variables in the model are held constant. We can also see that the education of the head has a positive effect at higher FCS levels and that the religion of the head and women having their own plots significantly affect FCS.

### 3.3. Recipes

**Figure 2:**
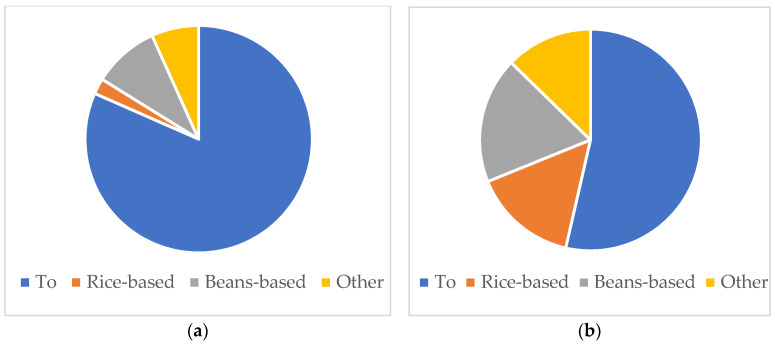
Average ratio of dishes of the day categorized by recipes, by commune (**a**) Yako and (**b**) Po. *To* is a stiff porridge made from millet, sorghum, or maize.

What do they usually eat? It is worth knowing the recipes when we consider people’s dietary habits and nutrition. From 24 h recall, we identify the recipes prepared on the previous day. To see the regional difference from the perspective of dishes, we illustrated the recipes prepared by the households on the previous day (Figure 2). For example, if the household prepared *To* (stiff porridge) and boiled cowpea during the day, the ratio was calculated as 0.5 *To* and 0.5 Beans-based. Figure 2 shows the average of all rounds. *To* is a very popular dish in Burkina Faso, made either from millet, sorghum, or maize, or a mixture. Rice-based dishes include recipes such as rice porridge, rice with sombala, and fried rice. Bean-based dishes include recipes such as boiled cowpea and fried beans. The regional difference is discernible here. *To* is the most popular dish both in Yako and Po; however, people in Po consume a wider variety of dishes.

### 3.4. Nutrient Supply

As FCS has not been validated to explain the nutrient quality dimension, we calculated nutrient supply per capita based on 24-h recall to analyze nutrient supply. The correlation between FCS and each nutrient was positive; for example, the correlation between FCS and energy was 0.34. In Table 4, the statistics are shown by communes, as we observed that regional differences were not negligible. The EAR of a representative individual and the ADER are presented in the table for reference. We cannot merely compare the mean to assess the degree of nutrient deficiency/excess supply due to the skewness of nutrient intake distribution, but apparently, most of the supply appeared to be below what was required, although the mean supply of carbohydrates and iron exceeded the appropriate requirements.

We also explored regional differences. We conducted a skewness test and found that no nutrient supply could be considered normally distributed. Hence, we conducted a Mann–Whitney U test, a test for nonparametric distributions, to ascertain whether the nutrient supply in these two communes differed significantly. The p-values generated from the test are presented in Table 4. The regional differences in the supply of energy, carbohydrates, proteins, zinc, vitamin C, thiamin, niacin, vitamin B6, folate, and vitamin B12 were statistically significant. In general, the nutrient supply was larger in Po (south) than in Yako (north). In contrast, the supply of iron was larger in Yako.

### 3.5. Macronutrient Balance

**Figure 3:**
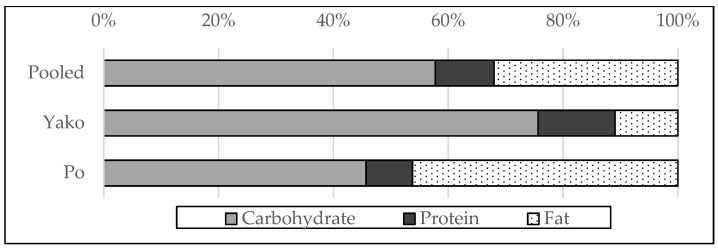
Percentage of macronutrient intake relative to total energy intake.

The percentage of macronutrient intake to total energy intake is shown in Figure 3. As mentioned in the Methods section, the AMDR is expressed as a percentage of total energy intake. An acceptable macronutrient balance to reduce the risk of disease is such that the percentages of energy from carbohydrates, protein, and fat ranged from 45–65%, 10–35%, and 20–35%, respectively. In the pooled sample, carbohydrates, proteins, and fat accounted for 58%, 10%, and 32% of energy intake, respectively, which seemed appropriate. However, we also noticed regional differences. While approximately three-quarters (76%) of energy intake was generated from carbohydrates in Yako, this proportion was less than half (46%) in Po. In Po, carbohydrates and fats were two major energy sources, while the percentage of energy intake was almost the same, approximately 46%. According to the AMDR, the macronutrient balance was not adequate for either region: Excessive amounts of carbohydrates and inadequate fat were consumed in Yako, whereas excessive amounts of fat and inadequate protein were consumed in Po.

### 3.6. Food Groups Contributing to Nutrient Supply

Next, we focus on the contribution of each food group to nutrient supply. The food items consumed in the previous 24 h were categorized into seven food groups: Cereals, roots/tubers, legumes, fruits/vegetables, meat/fish/eggs, fat/oil, and others. We investigated the source of energy, macronutrients (protein, carbohydrates, and fat), and several important essential micronutrients (calcium, iron, zinc, vitamin A, thiamin, riboflavin, niacin, vitamin B6, folate, and vitamin B12).

**Figure 4:**
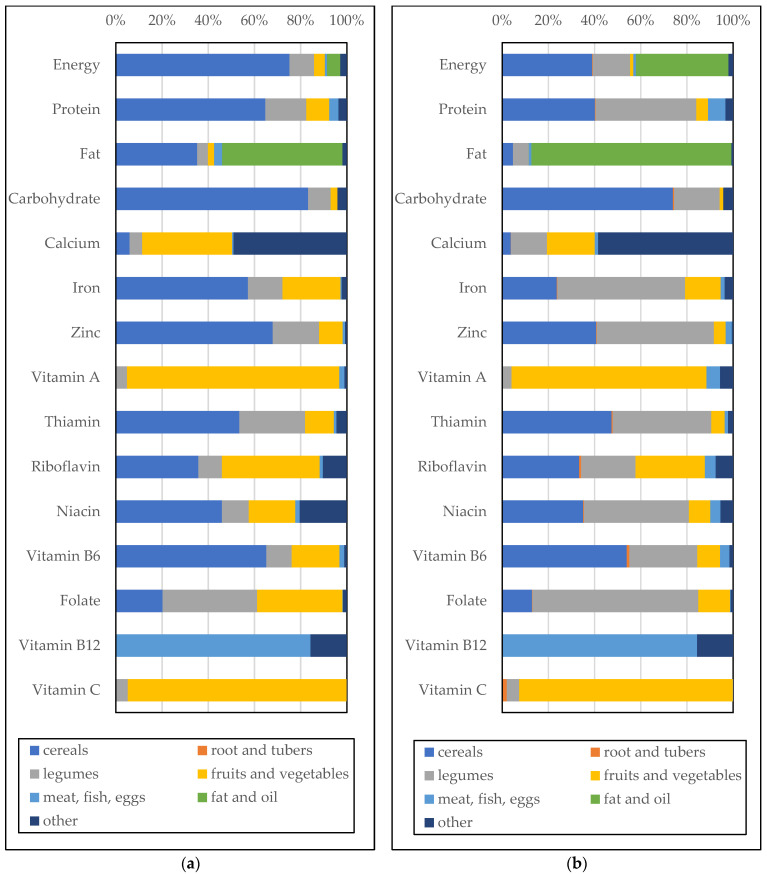
Nutrient source by food categories by region (**a**) Yako (**b**) Po.

Figure 4 shows the nutrient sources of food groups by commune. Cereals were the main source of nutrients, especially carbohydrates. Legumes were a significant source of proteins, many minerals, and various vitamins. Additionally, fruits and vegetables were the main nutrient sources of vitamins A and C. Similar to the macronutrient balance, we found regional differences in the nutrient sources. In Yako, three-quarters of energy intake was from cereals. In contrast, fat and oil exceeded cereals in terms of energy supply in Po; fat and oil constituted 40% and cereals constituted 39% of the energy supply.

## 4. Discussion

From the descriptive statistics, we noticed non-negligible regional differences between the northern (Yako) and southern (Po) parts of Burkina Faso, even though we selected comparable villages in terms of population and distance from the road and from the city center. The differences include religion, ethnicity, spoken language, and field size. Households in Po are more likely to have larger fields and cultivate a greater variety of crops. It is likely because the south experiences more favorable climatic conditions, such as more rainfall, which allows the growth of a wider variety of crops. Furthermore, Po’s geographical advantage of sharing a border with Ghana brings expanded trading opportunities and could incentivize farmers.

We presented the FCS as a part of the food security measure. Food security varies according to differences in household characteristics, region, and season. Significant regional differences were due to the more favorable situation in Po. The effects of the season on FCS were significant, although the magnitudes were smaller than the regional differences. Seasonal differences were primarily caused by changes in food availability due to the harvest period so it might be suggested to consider changing/introducing crops to grow or staggering planting time to smooth nutritional intakes throughout the year. We found that 2019 was a good harvest year because of unusually high rainfall. National cereal production in Burkina Faso increased by 12% above the average of the last five years, except in conflict-affected zones in 2019 [11]. Usually, market prices of the main crops increase during the lean season, resulting in seasonal fluctuations; however, prices in the 2019 lean season were lower than in the previous year. Hence, the seasonal fluctuation pattern may have been attenuated, compared to normal years. From the result of the ordered logit regression, significant effects of the head’s education and women’s plots imply not only geographical condition but also operations through knowledge or women’s empowerment could play important roles in food security.

Macronutrient balance calculated from 24-h recall seemed to be acceptable for the pooled sample, but when we considered region-specific macronutrient balance, values fell into an unacceptable range. While cereal was the dominant nutrient source in Yako, fat and oil played a considerable role as nutrient sources in Po. As Po had more advantageous climate conditions and trading opportunities, farmers in Po tended to be more affluent. This may support Bennett’s law [22], which states that people shift from staple foods to other foods (including meat, oils, and vegetables) as their income grows. It is said that an increase in income could lead to obesity or chronic diseases without nutritional improvement and a good food environment [23]; Po may be following this trend.

Most nutrient supplies seemed to be insufficient. Vitamin A is necessary for growth and development, maintenance of the immune system, and good vision, but vitamin A deficiency is serious in this sample. Cereals and oils, which are commonly consumed in these areas, are not good sources of vitamin A. Animal-source food and vegetables are generally rich in vitamin A, and farmers in this study derived vitamin A mostly from vegetables. Therefore, more animal-source food or vegetable consumption is recommended for the studied regions. In these areas, biofortification may also contribute to vitamin A deficiency. According to HarvestPlus [24], vitamin-A-fortified maize and orange sweet potatoes are being tested or released in Burkina Faso. Other micronutrient deficiencies included calcium, zinc, vitamin C, riboflavin, niacin, and vitamin B12. This is in line with a previous study in rural Burkina Faso [13] that showed that calcium, vitamin C, folate, and vitamin B12 were lower than the requirement for women and children. For nutrients assumed to have low bioavailability, such as zinc and iron, the absorption rate should be considered; however, there is no agreement on how to account for this [13]. Hence, we need to keep in mind that nutrient “intake” might be insufficient, even though nutrient “supply” seems to be sufficient.

We also investigated the source of nutrients. The characteristics that have not been well-documented but are distinctive in rural Burkina Faso may be the use of by-products of crops and *potash*. Rural farmers in Africa eat the leaves of crops such as cassava [25] and cowpea [8]. Although these are often seen as only by-products, they play an important role in the farmers’ nutritional supply. Some micronutrients are also provided from the “other” group, which contains condiments such as *potash*. *Potash* is an alkali obtained by leaching ashes with water or by purchasing it at a market; it is commonly used in cooking in Burkina Faso to add texture, shorten cooking time, and preserve food, among other purposes [26].

This study has some limitations. First, we divided food consumption by the number of household members who ate the meal to calculate per capita supply, without considering household composition. Hence, nutrient supply per capita per day is not sensitive to intra-household inequities in food consumption. Second, we did not analyze food consumed away from home. We adjusted the number of household members who ate away from home when we calculate the per capita consumption; however, missing information on what people eat away from home could result in an incomplete assessment of their nutrient supply. Third, the possibility remains that respondents may have failed to recall all the food and drink consumed, which may lead to an underestimation of their nutrient supply.

This study found that food security, nutrient supplies, recipes, and nutrient sources in Burkina Faso were subject to regional differences, seasonal changes, and household characteristics. Overall, we found large differences by region. It is said that a transition to a planetary health diet, an optimal diet for both people and the planet, was needed [27]. The planetary health diet is a global reference diet, which meets dietary needs while simultaneously decreasing environmental load. As a strategy in accordance with the planetary health diet, we need to change global food consumption. Simultaneously, local dietary environments vary by country and region. Hence, the implementation of this diet would require careful analysis of region-specific differences to achieve the best results.

Food security worldwide has been affected by the COVID-19 pandemic. Burkina Faso is no exception. Many factors, such as travel restrictions, fragmented supply chains, loss of household income, households’ limited coping mechanisms, and low levels of food assistance affected food security [11]. Vulnerable people, including rural farmers, have been the most affected by the pandemic. Although we could not assess the effect of COVID-19 or other global crises such as climate change and the recent Ukraine conflict in this survey, we would like to continue to pay attention to farmers’ food security and nutritional status in future research.

## Figures and Tables

**Table 1:** Food group weight for constructing the FCS.

Food Group	Examples	Weight
Main staples	Cereals, roots, and tubers	2
Pulses	Beans, peas, nuts	3
Vegetables	Vegetables, leaves	1
Fruit	Fruits	1
Meat and fish	Beef, pork, eggs, fish	4
Milk	Milk, dairy products	4
Sugar	Sugar, honey	0.5
Oil	Oil, fats, butter	0.5
Condiments	Spices, salt, coffee	0

Source: [14].

**Table 2:** Descriptive statistics per commune and pooled sample.

		Yako (% in Yako)		Po (% in Po)		Pooled (% in Pooled)	
Total number of households		670	-	506	-	1176	-
Village	Gobila	86	(12.8)		-	86	(7.3)
	Gollo	280	(41.8)		-	280	(23.8)
	Taonsgho	304	(45.4)		-	304	(25.9)
	Pinyiri		-	117	(23.1)	117	(9.9)
	Torem		-	239	(47.2)	239	(20.3)
	Adongo		-	150	(29.6)	150	(12.8)
Survey round	Apr 2019	116	(17.3)	97	(19.2)	213	(18.1)
	June 2019	119	(17.8)	92	(18.2)	211	(17.9)
	Aug 2019	116	(17.3)	71	(14.0)	187	(15.9)
	Oct 2019	115	(17.2)	78	(15.4)	193	(16.4)
	Dec 2019	105	(15.7)	80	(15.8)	185	(15.7)
	Feb 2020	99	(14.8)	88	(17.4)	187	(15.9)
Household size	mean (SD)	7.8	(±4.0)	6.5	(±2.9)	7.3	(±3.7)
Under five child ratio	% (SD)	11.8	(±15.6)	11.6	(±13.7)	11.7	(±14.8)
Age of head	mean (SD)	52.8	(±15.0)	50.5	(±16.8)	51.8	(±15.8)
Gender of head	Male	560	(83.6)	458	(90.5)	1018	(86.6)
	Female	110	(16.4)	48	(9.5)	158	(13.4)
Ethnicity of head	Gouroussi	0	(0.0)	400	(79.1)	400	(34.0)
	Mossi	670	(100.0)	75	(14.8)	745	(63.4)
	Other	0	(0.0)	31	(6.1)	31	(2.6)
Religion of head	Christian	431	(64.3)	201	(39.7)	632	(53.7)
	Muslim	187	(27.9)	245	(48.4)	432	(36.7)
	Animist	52	(7.8)	54	(10.7)	106	(9.0)
Education of head	Never attended school	572	(85.4)	331	(65.4)	903	(76.8)
	Attended school	98	(14.6)	175	(34.6)	273	(23.2)
Languages the head understands	Mossi	670	(100.0)	346	(68.4)	1016	(86.4)
(Multiple answers)	Gouroussi/Kassena	0	(0.0)	444	(87.7)	444	(37.8)
	French	6	(0.9)	155	(30.6)	161	(13.7)
	Ashanti	0	(0.0)	45	(8.9)	45	(3.8)
	Dioula	6	(0.9)	26	(5.1)	32	(2.7)
	Other	0	(0.0)	35	(6.9)	35	(3.0)
Women’s plot	Yes	188	(28.1)	71	(14.0)	259	(22.0)
	No	482	(71.9)	435	(86.0)	917	(78.0)
Field size (ha)	mean (SD)	1.9	(±1.1)	4.6	(±2.6)	3.0	(±2.3)
Number of crops grown in 2019	mean (SD)	3.1	(±1.1)	4.7	(±1.5)	3.8	(±1.5)

Note: Values are the number of households and percentages unless otherwise noted.

**Table 3:** Regression results from ordered logit model for FCS.

	Coef.	Std. Err.	*p*-Value
Season (base = Pre-harvest: June & August)			
Middle (February & April)	0.29	0.15	0.06 **
Post-Harvest (October & December)	0.48	0.16	0.00 ***
Commune (1 = Yako, 0 = Po)	−3.11	0.39	0.00 ***
Gender of Head (1 = Male, 0 = Female)	−0.17	0.22	0.43
Age of Head	0.04	0.03	0.15
Age of Head Squared	0.00	0.00	0.11
Religion of Head (base = Other)			
Muslim	0.80	0.20	0.00 ***
Christian	0.31	0.17	0.07 **
Ethnicity of Head (base = Other)			
Mossi	−0.09	0.53	0.87
Gouroussi	−0.17	0.43	0.70
Education of Head (1 = Attended, 0 = Never attended school)	0.43	0.19	0.03 **
Household Size	−0.03	0.02	0.22
Under 5 Child Ratio	−0.64	0.47	0.17
Number of crops grown in 2019	−0.02	0.06	0.73
Women’s plot (1 = Yes, 0 = No)	0.60	0.21	0.01 ***
/Cut1	−2.46	0.82	
/Cut2	−0.94	0.82	
Log likelihood Function	−910.59		
Wald Chi-Squared	465.28		
Pseudo R^2^	0.23		
Number of Observations	1175		

Significance is shown as: ** at the 5% level and *** at the 1% level.

**Table 4:** Mean values of nutrient supply by commune and requirements.

	Yako		Po		*p*-Value	Pooled		EAR	Cor. with FCS
	Mean	SD	Mean	SD	Yako & Po	Mean	SD		
Energy (kcal)	778.2	537.9	1529.8	922.0	(0.000)	1101.6	817.8	2225 ^(i)^	0.34
Macronutrients									
Carbohydrate (g)	141.7	91.5	168.6	69.6	(0.000)	146.1	153.2	83.8	0.21
Protein (g)	26.1	22.1	31.0	18.1	(0.000)	28.2	20.6	40.9	0.16
Micronutrients									
Calcium (mg)	386.4	620.0	390.7	395.8	(0.217)	388.2	535.0	800.0	0.10
Iron (mg)	11.7	18.4	9.6	7.8	(0.000)	10.8	14.9	7.1	0.03
Zinc (mg)	4.8	3.6	5.7	4.0	(0.000)	5.2	3.8	8.1	0.13
Vitamin C (mg)	20.0	22.6	22.5	20.5	(0.007)	21.1	21.7	67.5	0.10
Thiamin (mg)	0.7	0.8	1.1	0.7	(0.000)	0.9	0.8	1.0	0.21
Riboflavin (mg)	0.5	1.1	0.4	0.3	(0.112)	0.4	0.8	1.0	0.01
Niacin (mg)	5.2	18.9	6.3	4.7	(0.000)	5.7	14.6	11.5	0.12
Vitamin B6 (mg)	0.9	1.0	1.0	0.5	(0.000)	0.9	0.8	1.1	0.13
Folate (μg)	212.4	385.1	340.0	345.4	(0.000)	267.3	373.7	320.0	0.14
Vitamin B12 (μg)	0.2	0.2	0.3	0.5	(0.000)	0.2	0.4	2.0	0.20
Vitamin A (μgRAE)	60.8	99.3	53.4	64.4	(0.243)	57.6	86.1	562.5	0.10

^(i)^ EAR is shown as a reference requirement. For energy, ADER was used instead of the EAR.

## Data Availability

The data presented in this study are available from the corresponding author upon reasonable request.
